# Progress in 7SK ribonucleoprotein structural biology

**DOI:** 10.3389/fmolb.2023.1154622

**Published:** 2023-03-27

**Authors:** Momodou B. Camara, Amr M. Sobeh, Catherine D. Eichhorn

**Affiliations:** ^1^ Department of Chemistry, University of Nebraska, Lincoln, NE, United States; ^2^ Nebraska Center for Integrated Biomolecular Communication, Lincoln, NE, United States

**Keywords:** cryoEM, solution state NMR, X-ray crystallography, RNA structural dynamics, chemical probing, RNA-protein interactions, gene regulation

## Abstract

The 7SK ribonucleoprotein (RNP) is a dynamic and multifunctional regulator of RNA Polymerase II (RNAPII) transcription in metazoa. Comprised of the non-coding 7SK RNA, core proteins, and numerous accessory proteins, the most well-known 7SK RNP function is the sequestration and inactivation of the positive transcription elongation factor b (P-TEFb). More recently, 7SK RNP has been shown to regulate RNAPII transcription through P-TEFb-independent pathways. Due to its fundamental role in cellular function, dysregulation has been linked with human diseases including cancers, heart disease, developmental disorders, and viral infection. Significant advances in 7SK RNP structural biology have improved our understanding of 7SK RNP assembly and function. Here, we review progress in understanding the structural basis of 7SK RNA folding, biogenesis, and RNP assembly.

## Introduction

**FIGURE 1:**
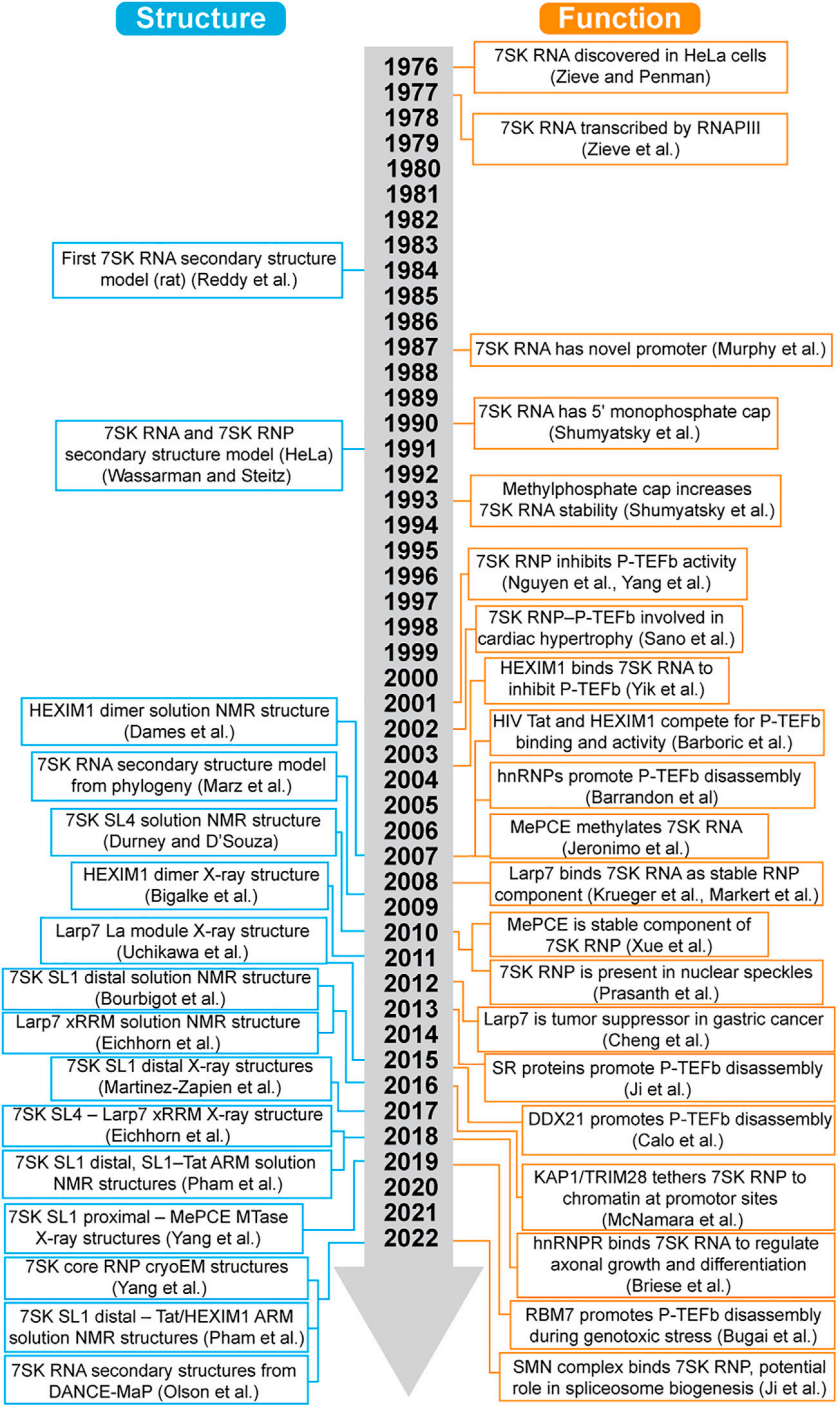
Timeline of advances in 7SK RNP structural biology and function.

7SK RNA is a highly abundant non-coding RNA with an estimated 2 × 10^5^ copies per cell (Gurney and Eliceiri, 1980), first identified in 1976 when it sedimented in the 7S fraction (Zieve and Penman, 1976). However, a specific function was not identified until 2001, when two research groups independently reported that 7SK RNA interacts with the positive elongation factor b (P-TEFb) to regulate RNA polymerase II (RNAPII) transcription elongation (Nguyen et al., 2001; Yang et al., 2001) (Figure 1). Shortly after, hexamethylene bis-acetamide (HMBA) induced in human vascular smooth muscle cells 1 (HEXIM1, also known as MAQ1 and CLP1) was shown to be required to recruit P-TEFb to the 7SK ribonucleoprotein (RNP) (Michels et al., 2003; Yik et al., 2003) (Figure 1). Core proteins methylphosphate capping enzyme (MePCE, also known as BCDIN3) and La-related protein 7 (Larp7, also known as PIP7S and HDCMA18P) were not identified until 2007 (Jeronimo et al., 2007) and 2008 (Krueger et al., 2008; Markert et al., 2008), respectively (Table 1). The first high-resolution structure of a domain component of 7SK RNA was reported in 2010 (Durney and D'Souza, 2010), two decades after establishing the secondary structure (Reddy et al., 1984; Wassarman and Steitz, 1991).

**TABLE 1:** 7SK RNP interacting proteins and associated functions.

Function	Protein/complex	Name	Association with 7SK RNP	References
Core proteins				
7SK RNA capping	MePCE	Methyl phosphate capping enzyme	SL1, Larp7	Jeronimo et al. (2007)
7SK RNP stabilization	Larp7	La-related protein 7	SL4, 3′ end, MePCE, P-TEFb	He et al. (2008), Krueger et al. (2008), Uchikawa et al. (2015)
Accessory proteins				
P-TEFb kinase activity inhibition	HEXIM1/2	Hexamethylene bisacetamide-induced protein 1 or 2	SL1, P-TEFb	Yik et al. (2003), Michels et al. (2004)
RNAPII regulation	P-TEFb	Positive transcription elongation factor b	HEXIM1, Larp7	Nguyen et al. (2001), Yang et al. (2001)
P-TEFb release	hnRNP A1, A2, B1, R, K, Q	Heterogeneous nuclear ribonucleoprotein A1, A2/B1, R, K, Q	SL3	Barrandon et al. (2007), Van Herreweghe et al. (2007), Briese et al. (2018)
P-TEFb release *via* CDK9 T186 dephosphorylation	PPM1α, PP2B, PPM1G	Protein phosphatase 1 α, γ, 2B	7SK RNA, P-TEFb	Chen et al. (2008), Gudipaty et al. (2015)
P-TEFb release	SRSF2	Serine/arginine-rich splicing factor 2	SL3	Ji et al. (2013)
P-TEFb release through helicase activity	DDX5, 6, 9, 21	DEAD box protein 5, 6, 9, 21	unknown	Van Herreweghe et al. (2007), Calo et al. (2015), Muck et al. (2016), Sithole et al. (2020)
P-TEFb release during cell stress	RBM7	RNA-binding motif 7 protein	SL3	Bugai et al. (2019)
P-TEFb dissociation from 7SK RNP and/or HEXIM1, kinase activation	Brd4	Bromodomain-containing protein 4	unknown	Jang et al. (2005), Schroder et al. (2012)
P-TEFb release by MePCE cleavage, 7SK RNA uncapping	JMJD6	Jumonji C-domain-containing protein 6	MePCE, P-TEFb, 5′ end	Liu et al. (2013), Lee et al. (2020)
Chromatin remodeling, enhancer RNA transcription	BAF	BRG1/BRM-associated factor	SL3	Flynn et al. (2016)
Transcription regulation of snRNA and snoRNA genes	LEC	Little elongation complex	unknown	Egloff et al. (2017)
P-TEFb binding enhancement to Tat	AFF1	AF4/FMR2 protein 1	Cyclin T1, Larp7, HEXIM1	Lu et al. (2014)
7SK RNP tethering to chromatin	KAP1	Kruppel-associated box (KRAM)-interacting protein 1	Larp7	McNamara et al. (2016b), McNamara et al. (2016c)
snRNP biogenesis regulation *via* association with 7SK-hnRNPs complex	SMN	Survival motor neuron	Larp7, MePCE	Ji et al. (2021)
7SK RNA pseudouridylation	DKC1	Dyskerin	SL3 (U250)	Zhao et al. (2016)
7SK RNA m6A modification	METTL3, METTL16	Methyltransferase-like protein 3, 16	7SK RNA, Larp7, MePCE	Warda et al. (2017), Covelo-Molares et al. (2021), Leger et al. (2021)

The past decade has seen a dramatic increase in high-resolution structures determined of domains of 7SK RNA, individual core and accessory proteins, and RNA-protein complexes (Table 2). Likewise, rapid developments in chemical mapping techniques and ensemble modeling approaches have provided a wealth of data on 7SK RNA secondary structure. These advances in 7SK RNP structural biology have provided a growing understanding of 7SK RNA structure and protein assembly to form a functional RNP. However, in the cellular environment a heterogeneous mixture of pools of 7SK RNPs are present, comprised of different sets of proteins each of which has a unique regulatory function (Table 1). This complexity challenges a detailed understanding of the structural basis for transcriptional regulation. Several excellent reviews discuss 7SK RNP functions and mechanisms in gene expression regulation (Peterlin et al., 2012; McNamara et al., 2016a; Briese and Sendtner, 2021; Studniarek et al., 2021; Fujinaga et al., 2022). Here, we describe progress in 7SK RNP structural biology, discuss outstanding challenges, and present perspectives for the future.

**TABLE 2:** Experimentally determined structures of 7SK RNP domains.

PDB ID	RNA domain(s)	Protein domain(s)	Year released	Method	Resolution	Comments	References
RNA							
2KX8	SL4	n/a	2010	NMR	n/a	Bound to arginine	Durney and D’Souza (2010)
5IEM	SL1 distal	n/a	2016	NMR	n/a		Bourbigot et al. (2016)
5LYS	SL1 distal	n/a	2017	X-ray	2.32 Å	Gold derivative	Martinez-Zapien et al. (2017)
5LYU	SL1 distal	n/a	2017	X-ray	2.20 Å		Martinez-Zapien et al. (2017)
5LYV	SL1 distal	n/a	2017	X-ray	2.35 Å	Osmium derivative	Martinez-Zapien et al. (2017)
6MCI	SL1 distal	n/a	2018	NMR	n/a		Pham et al. (2018)
6MCF	SL1 distal	Tat ARM	2018	NMR	n/a	Peptide sequence: GISYGRKKRRQRRRAHQ	Pham et al. (2018)
7T1N	SL1 distal	HEXIM1 ARM	2022	NMR	n/a	Peptide sequence: GISYGRQLGKKKHRRRAHQ	Pham et al. (2022)
7T1O	SL1 distal	Tat subtype G ARM	2022	NMR	n/a	Peptide sequence: GISYGRKKRRHRRRAHQ	Pham et al. (2022)
7T1P	SL1 distal	Tat Finland ARM	2022	NMR	n/a	Peptide sequence: GISYGRKKRKHRRRAHQ	Pham et al. (2022)
Core RNP							
5UNA	n/a	MePCE MTase	2017	X-ray	2.55 Å	Initial deposition 3G07 in 2009	n/a
4WKR	3′ UUU-OH	Larp7 La module	2015	X-ray	3.20 Å	construct residues 1-208	Uchikawa et al. (2015)
5KNW	n/a	Larp7 xRRM2	2016	NMR	n/a		Eichhorn et al. (2016)
6D12	SL4 upper stem	Larp7 xRRM2	2018	X-ray	2.20 Å		Eichhorn et al. (2018)
6DCB	SL1 proximal	MePCE MTase	2019	X-ray	2.00 Å	Substrate RNA	Yang et al. (2019)
6DCC	SL1 proximal	MePCE MTase	2019	X-ray	2.10 Å	Capped product RNA	Yang et al. (2019)
7SLP	Minimal 7SK SL1+SL4	Larp7, MePCE MTase	2022	CryoEM	4.10 Å	“Linear” model	Yang et al. (2022)
7SLQ	Minimal 7SK SL1+SL4	Larp7, MePCE MTase	2022	CryoEM	3.70 Å	“Circular” model	Yang et al. (2022)
P-TEFb assembly							
2GD7	n/a	HEXIM1	2007	NMR	n/a		Dames et al. (2007)
3S9G	n/a	HEXIM1 coiled-coil	2011	X-ray	2.10 Å	Δstammer	Bigalke et al. (2011)

## 7SK RNP: A master regulator of eukaryotic transcription

**FIGURE 2:**
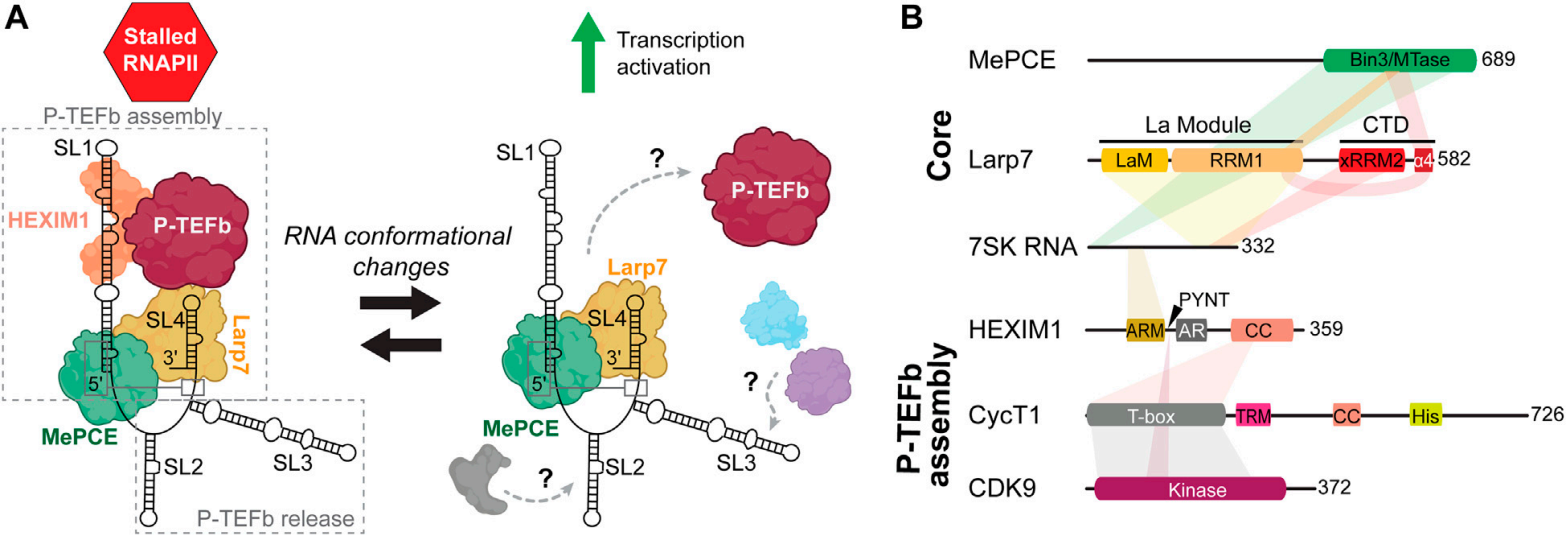
Overview of 7SK RNP components involved in P-TEFb regulation. **(A)** Cartoon schematic of P-TEFb assembly and release. Gray, blue, and purple assemblies indicate proteins involved in 7SK RNA remodeling and P-TEFb release (Table 1). **(B)** Domain topologies of proteins required for P-TEFb assembly, with 7SK RNA and protein-protein interaction sites indicated. Domain and sequence information is for human 7SK RNA and proteins.

7SK RNA is transcribed by RNAPIII and is primarily retained in the nucleus (Zieve et al., 1977; Murphy et al., 1987). Phylogenetic studies have identified 7SK RNA in metazoa with high sequence conservation, particularly among vertebrates (Gursoy et al., 2000; Gruber et al., 2008; Marz et al., 2009). In humans, 7SK RNA is a 331–334 nucleotide (nt) transcript expressed by a single gene on chromosome 6 (Driscoll et al., 1994). 7SK RNA has several posttranscriptional modifications including pseudouridylation (Zhao et al., 2016) and m6A (Warda et al., 2017; Leger et al., 2021) (Table 1). The nascent 7SK RNA is capped by MePCE with a monomethyl group at the 5′ γ-phosphate (Gupta et al., 1990; Shumyatsky et al., 1990). MePCE associates at promoter sites of 7SK and U6 (its other primary substrate) genes, suggesting MePCE is proximally located for co-transcriptional capping (Jeronimo et al., 2007; Xue et al., 2010). Characteristic of RNAPIII transcripts, 7SK RNA has a UUU-OH 3′ terminus, and a small fraction of 7SK RNA associates with genuine La protein (Stefano, 1984; Van Herreweghe et al., 2007). A converging model of 7SK RNA biogenesis is that MePCE first binds and caps the nascent 7SK RNA; next, La binds to the 3′ end after transcription termination; subsequently, Larp7 displaces La (Bayfield et al., 2010; Hasler et al., 2021) (Figure 2). Both MePCE and Larp7 are required to protect 7SK RNA from degradation (He et al., 2008; Krueger et al., 2008; Xue et al., 2010). Together 7SK RNA, MePCE, and Larp7 comprise the constitutively assembled ternary complex, called the 7SK core RNP (Figure 2), that is resistant to exonucleases, proteases, and high salt concentrations (Xue et al., 2010).

To this 7SK core RNP, assembly of numerous accessory proteins guides discrete mechanisms of transcription regulation (Quaresma et al., 2016; Briese and Sendtner, 2021) (Table 1). The most well-established 7SK RNP function is its role in P-TEFb regulation. The pausing of RNAPII ∼20–60 nt downstream of the transcription start site is a major transcriptional checkpoint in metazoa, resulting in a stalled, transcriptionally inactive, RNAPII complex (Dollinger and Gilmour, 2021). P-TEFb, a heterodimer comprised of cyclin-dependent kinase 9 (CDK9) and cyclin T1 (CycT1), phosphorylates serine 2 in the C-terminal domain of RNAPII in addition to negative elongation factors to promote the transition of RNAPII from a paused to a productive elongating state (Bowman and Kelly, 2014; Jonkers and Lis, 2015). To perform this task, P-TEFb associates with proteins including bromodomain-containing protein 4 (Brd4) and is part of the super elongation complex (SEC) (Bres et al., 2008; Bacon and D'Orso, 2019). 7SK RNP acts as a negative regulator against RNAPII transcription elongation by sequestering P-TEFb in a catalytically inactive pool. To recruit P-TEFb to the 7SK RNP, HEXIM1 (or, less commonly, its paralog HEXIM2) is minimally required to first assemble onto 7SK core RNP (Yik et al., 2003; Yik et al., 2005). Given the important role in transcription regulation, dysregulation of 7SK RNP and/or P-TEFb has been linked to myriad human diseases including cancers (Cheng et al., 2012; Thorne et al., 2022), developmental disorders (Alazami et al., 2012; Ling and Sorrentino, 2015), heart disease (Sano et al., 2002; Krystof et al., 2010; Catalucci and Condorelli, 2013), and neurodegenerative disease (Santoro et al., 2016; Schneeberger et al., 2019).

50%–90% of P-TEFb is associated with 7SK RNP, depending on cell type (Peterlin and Price, 2006). The 7SK RNA 5′ and 3′ ends are required for P-TEFb assembly (Egloff et al., 2006), whereas central residues appear to be required for P-TEFb release (Van Herreweghe et al., 2007; Briese et al., 2018) (Figure 2A). HEXIM1 binds directly to the distal region of the 5′ SL1. On binding 7SK RNA, HEXIM1 is able to stably recruit P-TEFb and inactivate CDK9 kinase activity. P-TEFb release is a highly complex process with dozens of identified proteins, many of which are involved in RNA processing (Table 1) (Barrandon et al., 2007; Flynn et al., 2016; Egloff et al., 2017). P-TEFb release may require dismantling of the 7SK core RNP: in one study, MePCE was shown to be cleaved by JMJD6 as part of the P-TEFb release mechanism (Lee et al., 2020). Several of these proteins associate with SL3 (Ji et al., 2013; Bugai et al., 2019; Luo et al., 2021), and a 7SK RNA construct lacking SL3 was able to bind but not release P-TEFb (Van Herreweghe et al., 2007; Briese et al., 2018), suggesting that SL3 may act as a hub to recruit proteins for P-TEFb release (Figure 2A). Rather than act as a static scaffold, 7SK RNA undergoes secondary structural remodeling as part of the mechanism of P-TEFb release (Van Herreweghe et al., 2007; Krueger et al., 2010; Calo et al., 2015).

Poised for action, 7SK–P-TEFb complexes are tethered to chromatin at promoter and enhancer sites (Ji et al., 2013; Wu et al., 2013; McNamara et al., 2016a; Flynn et al., 2016). There is growing consensus that rather than act as a global transcription regulator, P-TEFb-stimulated gene activation is localized and coordinated by cellular cues such as proliferative status and/or environmental cues such as DNA damage, stress, or small molecules (e.g., flavopiridol) (McNamara et al., 2016a; Bugai et al., 2019; Studniarek et al., 2021). In addition, infectious agents leverage 7SK RNP to reprogram host cell transcription to evade detection and promote survival. The most well-established example is HIV-1, where the HIV transcriptional transactivator (Tat) protein competes with HEXIM1 to bind 7SK RNA and recruit the 7SK–P-TEFb complex to the nascently transcribing HIV RNA genome (He and Zhou, 2011). Other infectious agents including SARS-CoV-2 (Gordon et al., 2020) and bacteria such as *Legionella* (Von Dwingelo et al., 2019; Cordsmeier et al., 2022) have also been shown to maneuver 7SK RNP to reprogram the host transcriptome.

Beyond P-TEFb regulation, 7SK RNP has been shown to regulate RNAPII function in pathways independent of P-TEFb. For example, 7SK RNP has been identified to regulate transcription initiation of enhancer RNAs through association with the chromatin-remodeling BRG1/BRM-associated factor (BAF) complex (Flynn et al., 2016); transcription elongation of small nuclear and nucleolar RNAs, localized in Cajal bodies (Egloff et al., 2017); and axonal growth and differentiation in motoneurons, localized in the cytosol (Briese et al., 2018; Briese and Sendtner, 2021) (Table 1). However, the limited biochemical data and lack of high-resolution structures of 7SK RNP accessory proteins in complex with 7SK RNA or core proteins preclude a detailed understanding of 7SK RNP-mediated transcription regulation.

## 7SK RNA secondary and tertiary structure

### 7SK RNA secondary structure

**FIGURE 3:**
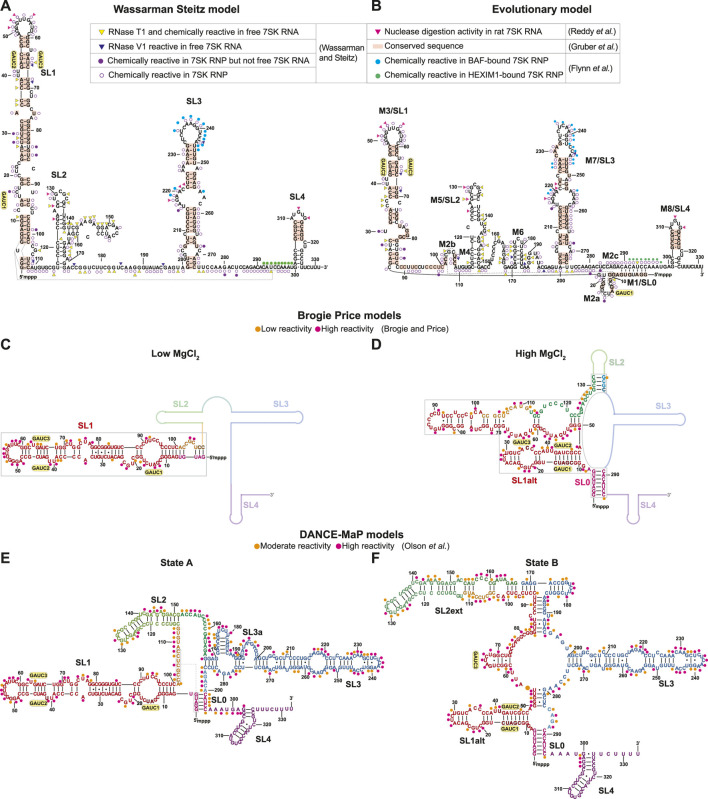
Secondary structure models of human 7SK RNA derived from biochemical and phylogenetic analysis. GAUC motifs are indicated by yellow circles. Residues are labeled according to evolutionary conservation (Gruber et al., 2008; Marz et al., 2009) and susceptibility to nuclease and/or chemical reactivity (Reddy et al., 1984; Wassarman and Steitz, 1991; Flynn et al., 2016; Olson et al., 2022). **(A)** Wassarman Steitz 7SK RNA secondary structure model (Wassarman and Steitz, 1991). **(B)** 7SK RNA secondary structure model from phylogenetic analysis (Marz et al., 2009). *Inset legend:* pink triangles show ssRNA-specific nuclease (RNase A1, RNase T1) cleavage activity in rat 7SK RNA (Reddy et al., 1984); red rectangles show conserved sequences in 7SK RNA (Gruber et al., 2008); yellow triangles show ssRNA-specific RNase T1 cleavage and chemical (DMS, Kethoxal and CMCT) modification in free 7SK RNA but not 7SK RNP; blue triangles show dsRNA-specific RNase V1 cleavage activity in free 7SK RNA but not 7SK RNP; filled purple circles show chemical modification sites in 7SK RNP but not free 7SK RNA; open purple circles show chemical modification sites in 7SK RNP (Wassarman and Steitz, 1991); filled cyan circles show in-cell SHAPE reactivity when BAF is bound to 7SK RNA; filled green circles show in-cell SHAPE reactivity when HEXIM1 is bound to the 7SK RNA (Flynn et al., 2016). **(C,D)** Secondary structure models of the 7SK RNA 5′ end in low and high MgCl_2_ concentration (Brogie and Price, 2017). Line representations indicate regions with unreported secondary structure. Orange and pink circles show low and high SHAPE reactivity, respectively. **(E,F)** Consensus secondary structure models of major states A and B, respectively, from DANCE-Map studies (Olson et al., 2022). Orange and pink circles show moderate and high DMS reactivity, respectively.

Several secondary structure models of 7SK RNA have been proposed based on experimental data from chemical probing techniques as well as evolutionary studies. Although differences are observed among studies, the 7SK RNA secondary structure generally consists of four stem-loop domains (SL1, SL2, SL3, SL4) with intervening single-stranded RNA (ssRNA) linker sequences. The first secondary structure model was reported in 1984, where 7SK RNA was extracted from rat cells and subjected to nuclease digestion (Reddy et al., 1984). Four regions of 7SK RNA were highly susceptible to nuclease activity: SL1 apical loop, SL2, SL3 internal loop, and SL4 apical loop, which were used in combination with maximal base-pairing to construct a secondary structure model (Figure 3A) (Reddy et al., 1984). A landmark study in 1991 (Wassarman and Steitz, 1991) used a combinatorial chemical and enzymatic probing approach with 7SK RNA samples isolated from HeLa cells in the presence and absence of at-the-time unknown protein cofactors. Overall, a “linear” secondary structure was identified consisting of four stem-loops (Figure 3A) that differed from the previous secondary structure model (Reddy et al., 1984) in SL1 and SL2 regions. Significant differences in reactivity were observed when comparing the 7SK RNA and 7SK RNP (Figure 3A). For example, SL1 distal, SL2, and SL2-SL3 linker regions were particularly reactive in free 7SK RNA but unreactive in 7SK RNP (Figure 3A). In contrast, SL1 proximal end and SL3 were reactive in 7SK RNP but unreactive in free 7SK RNA (Figure 3A). Here, differences in reactivity between 7SK RNA and 7SK RNP may be attributed to differences in secondary structure or protection from nuclease activity when bound to protein.

Sequence homology studies showed that SL1 and SL4 are highly conserved across both vertebrate and invertebrate 7SK RNA sequences (Figures 3A, B) (Gruber et al., 2008). The central stem-loops, particularly SL3, are conserved in vertebrates but less conserved in invertebrate species (Gruber et al., 2008; Yazbeck et al., 2018). A hallmark feature of 7SK RNA is the presence of 5′ GAUC sequences in SL1 distal (Egloff et al., 2006; Marz et al., 2009). This sequence was later found to be the binding site for HEXIM1/2 and HIV-1 Tat proteins (Lebars et al., 2010). Phylogenetic analysis of 7SK RNA sequences led to the proposal of a distinct secondary structure model, named ‘circular’ for a long-range base-pairing interaction between 5′ and 3′ residues to form SL0 (alternately called M1) (Figure 3B) (Marz et al., 2009). The “circular” model contains more stem-loops than the “linear” model, with multiple potential base-pairing arrangements at the proximal end of SL1 (Figure 3B). With the exception of the SL1 proximal end, this “circular” model shares similarities with the Wassarman Steitz “linear” model at SL1 distal end, SL3, and SL4 (Figures 3A, B), suggesting these regions are somewhat structurally conserved*.*

Recently, DMS and/or SHAPE chemical probing approaches have been used to investigate 7SK RNA secondary structure *in vitro* and *in cellulo* (Brogie and Price, 2017; Luo et al., 2021; Olson et al., 2022; Van Damme et al., 2022). Price and coworkers found that the 7SK RNA secondary structure, particularly SL1, was sensitive to MgCl_2_ concentration (Brogie and Price, 2017). In addition to the two GAUC motifs previously identified in SL1 distal end, a third GAUC motif near the 5′ end was identified (GAUC1, GAUC2, and GAUC3) allowing an alternate pairing arrangement (SL1alt) leading to the formation of a “circular” conformation at high MgCl_2_ concentrations (Figures 3C, D). The authors propose that 7SK RNA adopts a “linear conformation” in the presence of HEXIM1-P-TEFb and rearranges to the “circular” conformation on P-TEFb release (Brogie and Price, 2017). In a separate study, in-cell SHAPE experiments showed different chemical reactivities in 7SK RNA SL3 and SL3-SL4 linker regions when bound to HEXIM1 (P-TEFb dependent) or BAF (P-TEFb independent), suggesting different RNA secondary structures when bound to different protein cofactors (Figure 3A) (Flynn et al., 2016). Consistent with the model proposed by Price and coworkers, the chemical reactivities indicate that, in the HEXIM1-bound 7SK RNP, 7SK RNA adopts a “linear” conformation (Figures 3A,B). Additional secondary structure models in support of “linear” (Luo et al., 2021) or “circular” (Van Damme et al., 2022) conformations have been proposed using chemical mapping approaches.

Recent advances in chemical probing combined with ensemble detection methods have helped to untangle 7SK RNA secondary structure heterogeneity. Olson et al. (2022) used deconvolution and annotation of ribonucleic conformational ensembles (DANCE-MaP), a technique that relies on DMS modification and a maximum likelihood deconvolution strategy to generate secondary structure ensembles. These experiments revealed a structural ensemble of two major conformational states, where state A resembles the “linear” model and state B resembles the “circular” model (Figures 3E, F). Both A and B states contain the SL0 stem observed in the “circular” model (Figures 3E, F), although the SL0 stem in state A showed an increased reactivity relative to state B suggesting a “linear-like” conformation. The most significant difference between the two major states is found in SL1 and SL2. In state B, this region undergoes rearrangement resulting in the formation of an alternate SL1 (SL1alt) and an extended SL2 (SL2ext), which has not been previously observed in literature (Figure 3F). Interestingly, the populations of states A and B differed in different cell types and upon treatment of P-TEFb inhibitors, suggesting that 7SK RNA secondary structure is sensitive to cellular and environmental conditions. Together, the past three decades of biochemical and bioinformatics studies on 7SK RNA secondary structure have advanced understanding of 7SK RNA folding and begun to resolve ensembles of secondary structures, leading to models of secondary structure switching coupled to functional states.

### High-resolution structures of SL1

**FIGURE 4:**
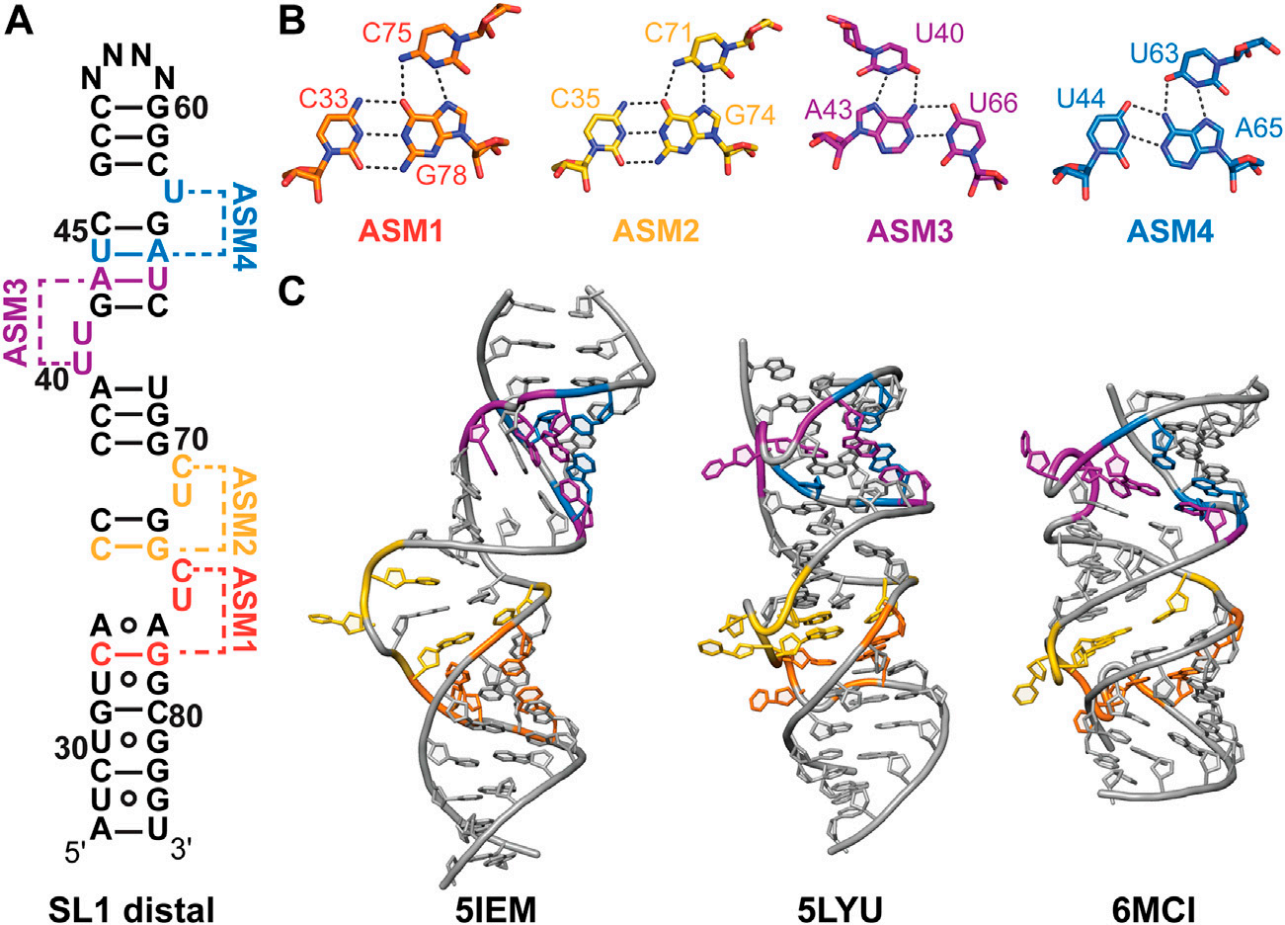
Structures of 7SK RNA SL1 distal region. **(A)** Secondary structure model of the SL1 distal constructs used to determine solution NMR or X-ray crystal structures. N indicates variable apical loop sequence. ASMs are shown as defined by D’Souza and coworkers (Pham et al., 2018). **(B)** Base triples of arginine sandwich motifs (ASM) for solution NMR structure (PDB ID 7T1N). **(C)** Structures of SL1 distal constructs from solution NMR (PDB IDs 5IEM, 6MCI) or X-ray crystallography (PDB ID 5LYU).

The highly conserved SL1 distal end (nts 27–84) has a near-identical secondary structure for experimentally and evolutionarily determined secondary structures (Figure 3). Several solution NMR and X-ray crystallographic structures have been determined for the SL1 distal region with a modified apical loop (Table 2; Figure 4). The SL1 distal secondary structure features several short stems separated by pyrimidine-rich asymmetric internal loops capable of forming base triples with nearby base-pairs (Figures 4A, B). D’Souza and coworkers named this motif the “arginine sandwich motif” (ASM) (Figures 4A, 8B) (Pham et al., 2018). This motif has been previously observed in other biologically important RNAs, in particular the HIV-1 transactivation response (TAR) element (Tao et al., 1997; Brodsky et al., 1998; Huthoff et al., 2004).

Dock-Bregeon and coworkers determined one solution NMR structural ensemble and three X-ray crystal structures of a SL1 distal construct (Table 2) (Bourbigot et al., 2016; Martinez-Zapien et al., 2017). In the absence of divalent cations, the RNA adopts an extended conformation where base triples do not form (Figure 4C). In contrast, X-ray crystal structures determined in the presence of divalent cations revealed four coaxially stacked subdomains and one base triple at each loop (Figure 4C). Differences in base triple pairing arrangements were observed among structures obtained for native, osmium-, and gold-soaked crystals, indicating conformational variability at these sites. Independently, D’Souza and coworkers determined a solution NMR structure of a SL1 distal construct and observed formation of all ASM motifs (Figure 4C; Table 2) (Pham et al., 2018). Comparison of this solution NMR structure (PDB ID 6MCI) to the X-ray structure of native crystals (PDB ID 5LYU) show similar base triple formation for ASM1, ASM2, and ASM3. For ASM4, U_63_ forms a base triple to either G_42_-C_67_ (native crystal, PDB ID 5LYU) or U_44_-A_65_ (solution NMR, PDB ID 6MCI). A detailed comparison of X-ray and solution NMR structural differences is discussed elsewhere (Brillet et al., 2020).

All SL1 distal structures have globally similar conformations, although differences are observed in helical compaction, loop backbone conformation, and interhelical bending (Figure 4C). Molecular dynamics simulations indicate that ASM base triples are weak and transient (Roder et al., 2020). Sample conditions differed for all determined structures in particular pH, temperature, and presence of divalent cations. To reconcile these differences, Dock-Bregeon and coworkers examined the effect of pH and MgCl_2_ on SL1 distal conformation (Brillet et al., 2020). Small-angle X-ray scattering (SAXS) and NMR studies showed that addition of MgCl_2_ resulted in a more compact molecule, consistent with base triple formation. A metal binding site was identified in ASM3, which resulted in metal-induced bending about ASM3 on addition of MgCl_2_. Compaction was shown to be driven by addition of divalent cations or protonation of C_71_ (ASM2), C_75_ (ASM1), and A_77_ (ASM1) which may explain the observed structural differences.

### High-resolution structures of SL4

In addition to SL1, SL4 is highly conserved among metazoa and consists of a short stem-loop with a two-nt asymmetric internal loop (Figure 6A). A solution NMR structure was determined for a SL4 construct in complex with arginine (Figure 6B; Table 2) (Durney and D'Souza, 2010). In this arginine-bound structure at low pH, C_320_ in the C_320_U_321_ loop was found to form a base triple with the G_303_-C_323_ base-pair (Durney and D'Souza, 2010), similar to ASM motifs observed in SL1 described above. In a separate study, solution NMR relaxation experiments of SL4 showed that in the absence of amino acid or protein substrates the C_320_U_321_ loop is dynamic, suggesting that the base triple does not form (Eichhorn et al., 2018). An X-ray crystal structure of the SL4 upper stem (nts 305–319) bound to the Larp7 extended RNA recognition motif 2 (xRRM2) was determined (Eichhorn et al., 2018) (Table 2). When bound to Larp7, the SL4 upper stem was observed to have widening of the major groove, attributed to insertion of the xRRM2 helix α3 (Figure 6C). In addition, significant differences in the apical loop were observed compared to the previously determined NMR structure (Eichhorn et al., 2018) (Figures 6B, C). The apical loop backbone makes an S-turn about U_313_ and G_314_, stabilized by protein-RNA interactions. These structural features explain the high specificity of 7SK RNA recognition provided by the Larp7 xRRM2.

## 7SK core RNP

Prior to structural studies, several lines of biochemical evidence suggested that 7SK RNA, MePCE, and Larp7 engage in a complex interplay of interactions in the 7SK core RNP. The MePCE−Larp7 interaction is RNA-dependent: MePCE can be assembled on a Larp7−7SK RNA complex, even in the absence of a MePCE binding site, indicating that Larp7 is scaffolded onto 7SK RNA to provide a MePCE interaction surface (Muniz et al., 2013). Larp7 assembly results in loss of MePCE methyltransferase activity (Xue et al., 2010; Brogie and Price, 2017) through interactions with the C-terminal helix α4, also called the MePCE interacting domain (MID) (Figure 2B) (Brogie and Price, 2017). High-resolution structures of individual domains of core proteins in isolation or bound to 7SK RNA domains have provided critical insights into RNA recognition. Further, cryoEM structures of the 7SK core RNP ternary complex have aided understanding of 7SK RNA biogenesis and assembly into a stably associated core RNP (Yang et al., 2022).

### MePCE and MePCE−RNA

**FIGURE 5:**
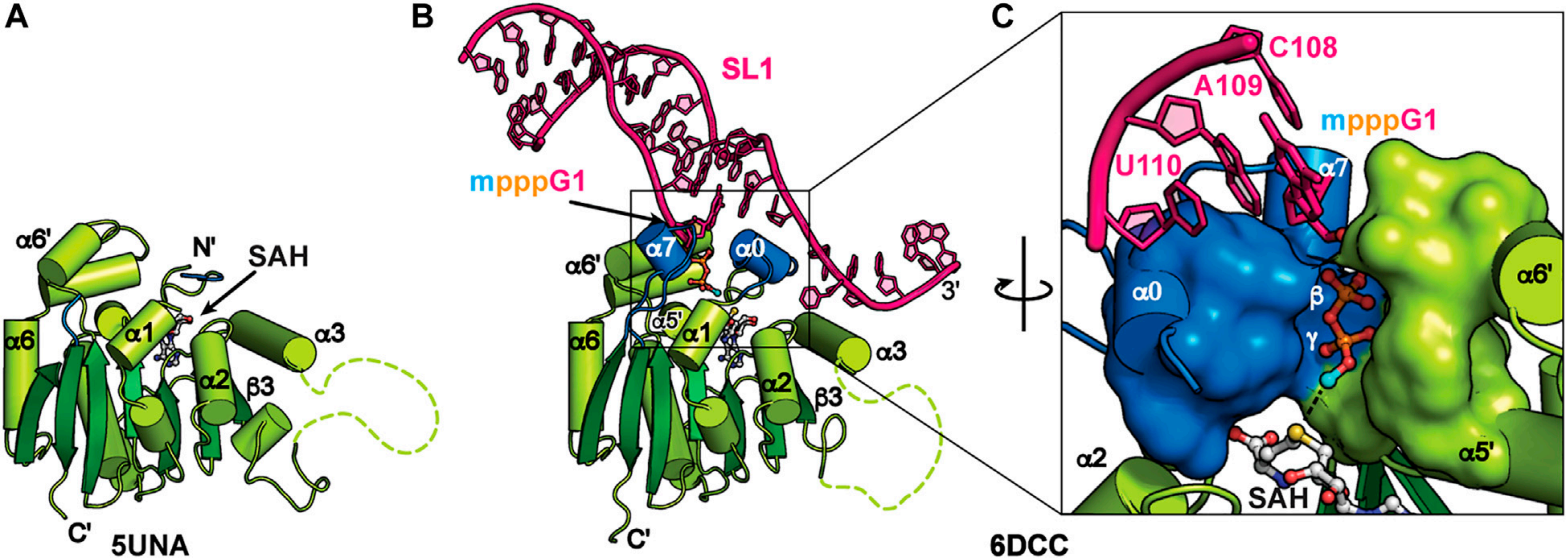
Structures of MePCE and MePCE-RNA. **(A)** X-ray crystal structure of the MePCE MTase domain, **(B)** X-ray crystal structure of MePCE MTase bound to a capped 7SK SL1 proximal construct, **(C)** The 5′ RNA triphosphate is secured in the MePCE MTase active site by a “triphosphate binding tunnel” (PDB ID 6DCC) shown in surface and cartoon representation. Helices α0 and α7, which form on RNA binding, are colored blue. Cofactor byproduct SAH and RNA 5′ triphosphate, terminal base pair and 3′ overhang are shown in ball and stick representation. Methyl cap is colored cyan.

MePCE is a mammalian ortholog of the *Drosophila* bicoid-interacting protein 3 (Bin3) (Jeronimo et al., 2007). In addition to 7SK RNA, MePCE methylates a subset of RNAPIII small nuclear RNAs including U6, B2, and U3 (Gupta et al., 1990; Shumyatsky et al., 1990). MePCE contains a variable N-terminal domain with predicted low structural complexity and a C-terminal methyltransferase (MTase) domain (Figure 2B) (Cosgrove et al., 2012). The MTase domain uses cofactor S-adenosyl methionine (SAM) as the methyl donor to transfer the methyl group to the RNA 5′ γ-phosphate. For efficient binding and catalysis, a terminal G-C base-pair and 3′ ssRNA overhang are required (Singh et al., 1990; Yang et al., 2019). An X-ray crystal structure of the human MTase domain in complex with S-adenosyl homocysteine (SAH) byproduct, determined by the Structural Genomics Consortium in Toronto (Table 2), identified a classical Rossman fold from residues 431–685 with a disordered α3-β3 loop (residues 492–538) (Figure 5A).

X-ray crystal structures of the human MePCE MTase domain in complex with RNA constructs of the 7SK RNA 5′ proximal hairpin revealed the structural basis for capping and retention after catalysis (Figure 5B) (Yang et al., 2019). Structures were determined of MTase-SAH bound to either uncapped substrate RNA or capped product RNA (Table 2). When bound to RNA, the MTase has substantial conformational changes surrounding the active site including ordering of N-terminal residues 411–430 to form new helix α0 (Figure 5). Helix α0 is enriched in tyrosine residues, which lie underneath the terminal base-pair in the RNA helix and interact with the RNA helix-overhang interface (Figures 5B, C). Residues in the β6-β7 loop become ordered, forming new helix α7, and interact with both RNA and N-terminal residues (Figures 5B, C). The RNA 5′ triphosphate is encased within the active site *via* a “triphosphate binding tunnel” comprised of residues in α0, α7, α5′, and α6′ (Figure 5C). An extensive network of hydrogen bonds fixes the 5′ triphosphate in place and positions the terminal oxygen on the 5′ γ-phosphate in line for methyl transfer (Figure 5C). Alanine substitution of residues in the triphosphate binding tunnel significantly impaired catalysis (Yang et al., 2019).

Kinetics experiments showed poor enzymatic turnover, which was explained by ITC binding experiments showing that the methylated RNA product had 3-fold improved binding affinity compared to substrate RNA. Catalytic turnover was found to be inhibited both by addition of SAH and methylated RNA (Yang et al., 2019). An RNA construct of the “linear” SL1 proximal hairpin and 3′ overhang sequence had higher affinity binding compared to an RNA construct of the “circular” SL0 hairpin and 3′ overhang sequence (Yang et al., 2019). Together, these structural and biochemical data explain how MePCE is retained on 7SK RNA after catalysis as part of the core 7SK RNP.

### Larp7 and Larp7−RNA

**FIGURE 6:**
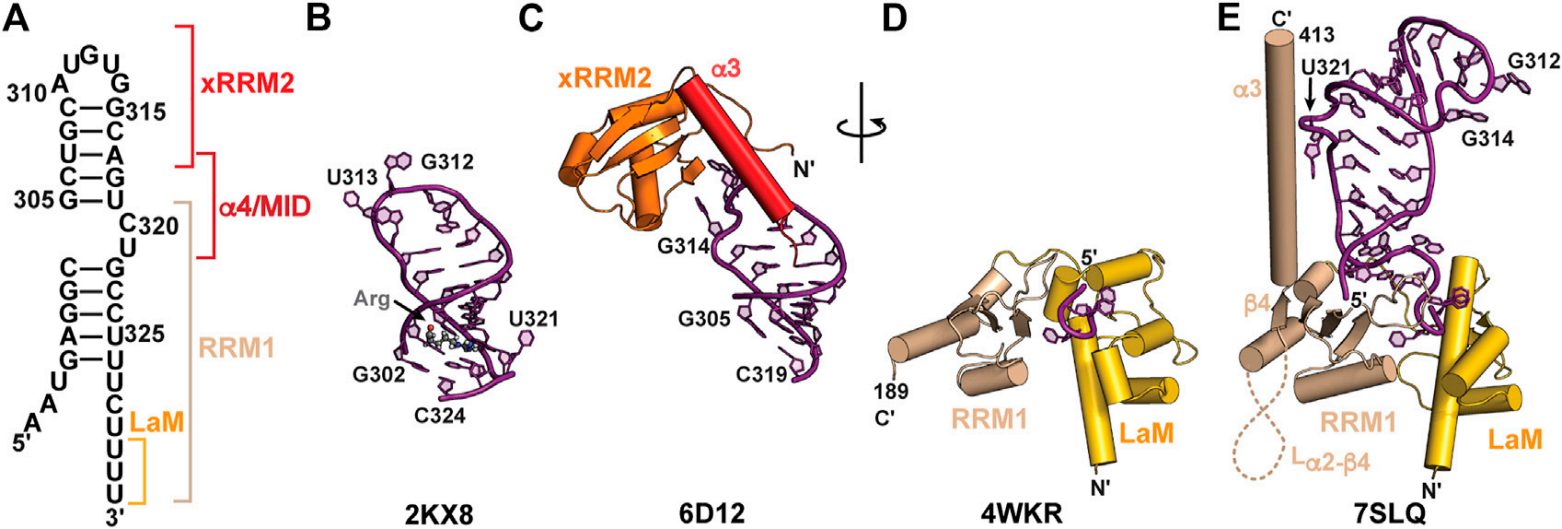
Structures of 7SK RNA 3′ end and Larp7 recognition of 7SK RNA. **(A)** Secondary structure model of the 7SK RNA 3′ end (nts 297–332), with Larp7 interaction sites indicated in brackets. **(B)** Solution NMR structure of 7SK SL4 bound to arginine (Arg). Arginine ligand is shown in stick representation. **(C)** X-ray crystal structure of Larp7 xRRM2 bound to SL4 upper stem. **(D)** X-ray crystal structure of Larp7 La module bound to UUU RNA sequence. **(E)** 7SK core RNP cryoEM structure (PDB ID 7SLQ) reveals cryptic β4 and α3 in Larp7 RRM1 and extensive La module-RNA binding surface.

Larp7 is a member of the LARP7 class of the La and La-related protein superfamily (Bayfield et al., 2010; Maraia et al., 2017). In ciliates and fission yeast, which lack 7SK RNA, LARP7 proteins bind to telomerase RNA to function in telomerase biogenesis and activity (Aigner and Cech, 2004; Singh et al., 2012; Collopy et al., 2018; Mennie et al., 2018; Paez-Moscoso et al., 2018). In metazoa, Larp7 primarily binds to 7SK RNA (He et al., 2008; Krueger et al., 2008; Markert et al., 2008) although more recently Larp7 has been shown to bind Lin28 mRNA (Dai et al., 2014) as well as U6 snRNA and U6-specific snoRNAs (Wang et al., 2020; Hasler et al., 2021). Larp7 has two RNA-binding domains: an N-terminal La module comprised of a La motif (LaM) followed by an RRM1 and C-terminal atypical xRRM2, with a variable-length intervening linker sequence (Figure 2B). The La module binds to the UUU-OH 3′ terminus whereas the xRRM2 binds to the SL4 apical loop and upper stem (Figure 6).

**FIGURE 7:**
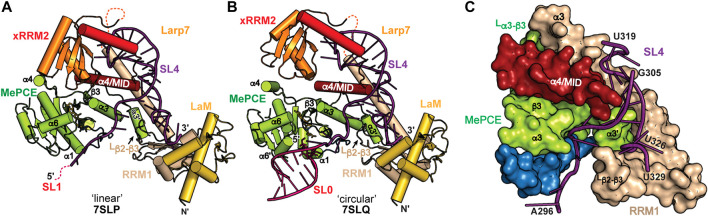
CryoEM structures of 7SK core RNP ternary complex. RNA constructs model the **(A)** “linear” or **(B)** “circular” conformations. **(C)** SL4–Larp7–MePCE interface features extensive interactions (PDB ID 7SLQ). MePCE MTase is colored green and residues corresponding to helix α0, which becomes unstructured in the ternary complex, are colored blue. Larp7 RRM1 is colored apricot and helix α4 is colored maroon.

An X-ray crystal structure of the human La module (construct residues 1–208) in complex with an RNA UUU trimer showed a similar RNA recognition mode to genuine La (Uchikawa et al., 2015) (Figure 6D; Table 2). RNA substrate is sandwiched between LaM and RRM1 domains, which are arranged in a “V” orientation, where the LaM consisted of a classical winged helix-turn-helix and the RRM1 contained three β-strands rather than the four-five typically observed in RRMs (Figure 6D). CryoEM structures of an *in vitro* reconstituted 7SK core RNP, which included recombinantly expressed full-length human Larp7, revealed new insights into Larp7 structure and recognition of 7SK RNA (Yang et al., 2022) (Table 2). The La module has a larger binding surface than previously observed for any La or LARP, in which the La module binds to all eight ssRNA nts. Aromatic residues in β2 and β3 strands on the RRM1 β-sheet surface interact with ssRNA nts. The RRM1 β2-β3 loop lies at the base of SL4 and contacts both the 5′ AAAU and 3′ ssRNA overhangs (Figures 6E, 7C). Surprisingly, the Larp7 RRM1 was found to contain a cryptic β4 strand and helix α3, with a 189-residue α2-β4 loop (Yang et al., 2022). Elegant NMR experiments demonstrated that the RRM1 β4 and α3 are stable components of the La module, even in the absence of RNA (Yang et al., 2022). Helix α3 is positioned perpendicular to the β-sheet and traverses up SL4 (Figure 6E). In contrast to the solution NMR structure of SL4 bound to arginine, the C_320_•G_303_-C_323_ base triple is not formed; rather, C_320_U_321_ loop residues are extruded to interact with RRM1 helix α3 (Yang et al., 2022), explaining biochemical assays demonstrating the importance of C_320_U_321_ loop residues for Larp7 recognition of 7SK RNA (Muniz et al., 2013; Uchikawa et al., 2015; Brogie and Price, 2017).

The C-terminal atypical xRRM2 is required for specific recognition of 7SK RNA and binds to the SL4 apical loop (Uchikawa et al., 2015; Eichhorn et al., 2018). The xRRM2 is a signature of LARP7 proteins, initially identified in the *Tetrahymena thermophila* telomerase protein p65 (Singh et al., 2012). A solution NMR structure of the human Larp7 xRRM2 showed a similar global structure to the *Tetrahymena* p65 xRRM2 (Eichhorn et al., 2016). Like canonical RRMs, the xRRM2 contains a βαββαβ topology with α-helices packing underneath an antiparallel β-sheet surface, which in canonical RRMs serves as the RNA binding surface. However, unlike canonical RRMs, the xRRM2 contains an additional helix α3 that is stably fixed atop the β-sheet, occluding this surface from RNA binding. In addition, the RRM-conserved RNP1 and RNP2 sequences on β3 and β1, respectively, are absent, and the xRRM2 contains a conserved RNP3 sequence on β2 (Singh et al., 2012; Basu et al., 2021). An X-ray crystal structure of the human Larp7 C-terminal domain showed that, consistent with the mode of xRRM2−RNA recognition, RNA bound to the side of the β-sheet rather than the surface (Figure 6C). Helix α3 has extensive contacts to RNA and lies along the major groove to direct the C-terminal residues down the RNA helical axis. Helix α3 residue W_533_ formed a stacking interaction with N-terminal residue F_451_ to position the N-terminus alongside helix α3 and down the SL4 helical axis (Figure 6C). This interaction may help to position the RRM1 helix α3 in close proximity to xRRM2. CryoEM structures of the 7SK core RNP showed that the xRRM2 interacts with the SL4 apical loop in the same manner as observed in the previously determined X-ray structure. The crystal construct lacked α4/MID residues, and in the cryoEM structure helix α4 crosses SL4 and engages in hydrophobic interactions with the Larp7 RRM1 helix α3 and MePCE MTase (Figure 7). Together, Larp7 interacts with the entire length of SL4, predominantly in the major groove and loops, explaining how Larp7 recognition of 7SK RNA protects the 3′ end from exonucleolytic cleavage and promotes Larp7-MePCE binding.

### MePCE−7SK RNA−Larp7 ternary complex

7SK RNA secondary structure heterogeneity has been a major obstacle to obtaining high-resolution structural information of 7SK RNP macromolecular complexes. One solution to this problem was presented by Feigon and coworkers (Yang et al., 2022). Minimal 7SK RNA constructs were rationally designed that included 5′ and 3′ regions, required to bind MePCE and Larp7, and lacked the central RNA stem-loops. A similar SL1-SL4 construct was previously shown to be competent for P-TEFb assembly (Van Herreweghe et al., 2007; Briese et al., 2018), indicating this construct is biologically relevant. To address the question of “linear” and “circular” 7SK RNA secondary structure models, minimal 7SK RNA constructs representing either “linear” or “circular” models were *in vitro* reconstituted with recombinantly expressed human MePCE MTase and full-length human Larp7 to form minimal 7SK core RNP ternary complexes. CryoEM structures were determined for both complexes and found to be overall similar (Figures 7A, B), suggesting the core RNP can accommodate different 7SK RNA conformations (Yang et al., 2022). The most substantial difference is observed at the 5′ hairpin: in the “linear” 7SK core RNP SL1 showed no visible density and could not be modeled in the structure (Figure 7A) whereas in the “circular” 7SK core RNP SL0 had clear density and could be modeled (Figure 7B).

The ternary structures show distinct conformational differences when compared to individual domains, suggesting that 7SK core RNP assembly induces structural changes to both RNA and protein. RNA binding to Larp7 fixes the orientations of the three Larp7 domains (La module, xRRM2, α4/MID), providing a binding surface for MePCE−Larp7 interaction. In addition to protein-RNA interactions, there is an extensive MePCE−Larp7 interaction interface at MePCE α3-β3 loop, Larp7 RRM1 α3, and α4/MID (Figure 7C). For X-ray crystal structures of MePCE in the absence and presence of RNA, the 61 aa α3-β3 loop is disordered (Figures 5A, B). However, in the ternary complex an additional helix α3′ and ordered loop form to interact with Larp7 RRM1 α3 and α4/MID (Figure 7C). The previously determined X-ray crystal structure of the MePCE−SL1 complex (Yang et al., 2019) showed specific interactions with the terminal base-pair and 3′ ssRNA overhang (Figure 5C). In the representative “circular” 7SK RNP structure, MePCE also binds to the SL0 terminal base-pair and 3′ ssRNA overhang (Figures 7B, C). However, the RNA binding pocket is remodeled such that the triphosphate binding tunnel is disorganized, particularly helices α0 and α7, thereby disrupting active site geometry. These structures explain enzymatic inactivation of MePCE induced by Larp7 and provide the first structural insights into 7SK RNP macromolecular assembly.

## 7SK RNP in P-TEFb sequestration and inactivation

The prominence of P-TEFb in human health and disease has made it a promising therapeutic target for wide-ranging diseases such as cancer (Anshabo et al., 2021), cardiac hypertrophy (Krystof et al., 2010), and HIV-1 (Massari et al., 2013). Over two dozen X-ray crystal structures have been determined of P-TEFb, the majority of which are bound to small molecule inhibitors, HIV-1 Tat, or SEC domain components. More recently, cryoEM structures of the RNAPII-SEC complex have provided key insights into delivery of P-TEFb to RNAPII and mechanisms of transcription elongation (Schier and Taatjes, 2020; Chen et al., 2021). In sharp contrast, there are at present no structures of P-TEFb bound to any component of 7SK RNP. Here, we summarize current understanding of HEXIM1 structure, HEXIM1−7SK RNA recognition, and biochemical evidence for HEXIM1−P-TEFb recognition.

### HEXIM1 and HEXIM1−7SK RNA

**FIGURE 8:**
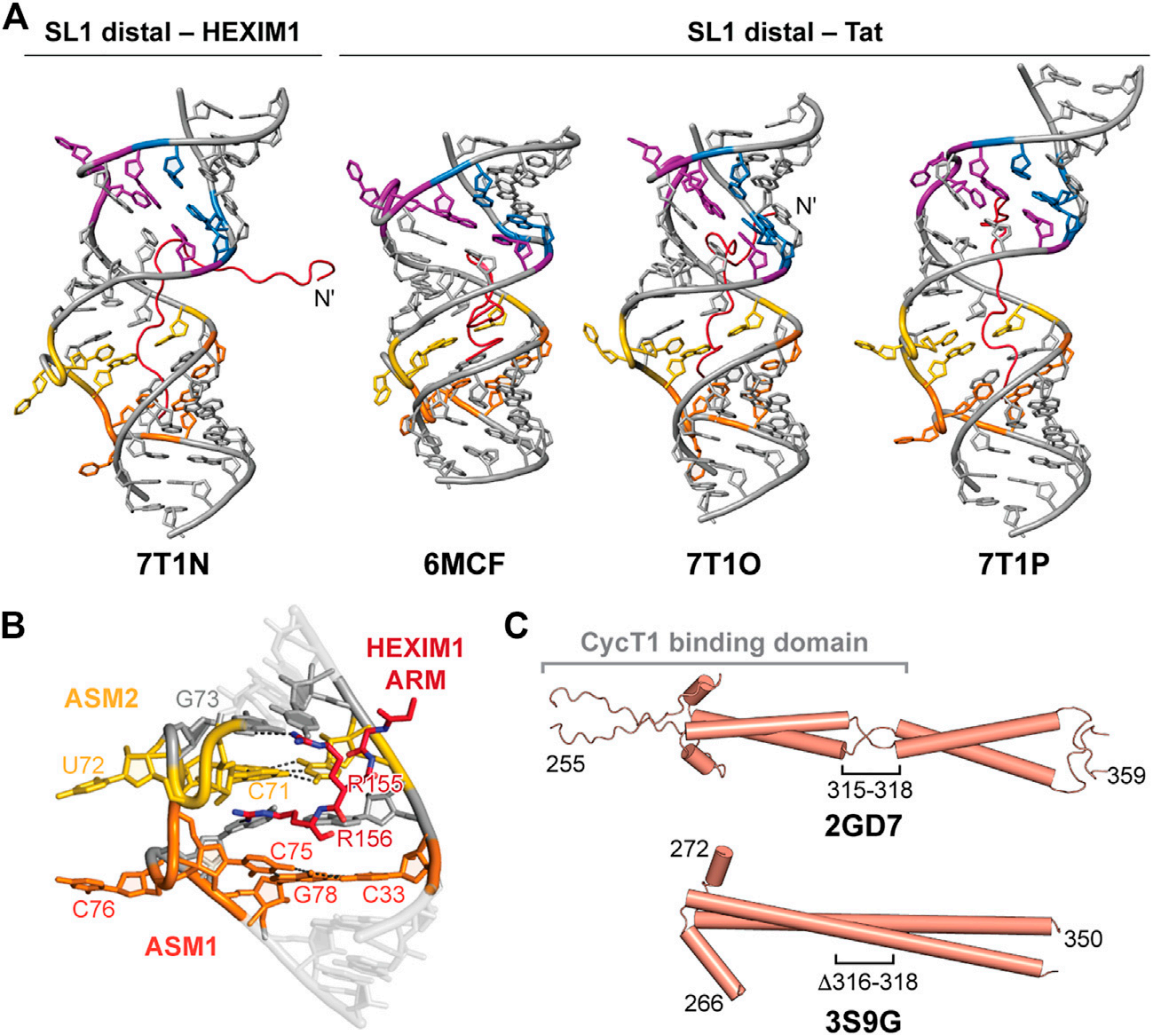
HEXIM1 and ARM recognition of 7SK SL1 RNA. **(A)** Solution NMR structures of SL1 distal bound to Tat or the first segment of the HEXIM1 ARM peptides. **(B)** ASM1-2 interacts with arginine residues 155–156 in HEXIM1 ARM (PDB ID 7T1N). **(C)** Solution NMR (PDB ID 2GD7) and X-ray crystal (PDB ID 3S9G) structures of HEXIM1 coiled-coil domain, with the putative CycT1 binding domain indicated in brackets.

HEXIM1 is a promiscuous double-stranded RNA (dsRNA) binding protein (Li et al., 2007; Michels and Bensaude, 2018) that regulates differentiation, inflammation, development, and heart physiology (Michels et al., 2004). HEXIM1/2 contains an unstructured N-terminus and coiled-coil domain at the C-terminus that mediates dimerization (Dames et al., 2007; Schonichen et al., 2010). HEXIM1 contains several functional domains: a variable N-terminal region (residues 1–120); basic region (BR), alternately called an arginine rich motif (ARM, residues 150–165), and entwined bipartite nuclear localization signal (NLS, residues 150–177) (Yik et al., 2004); conserved PYNT motif (residues 202–205); acidic region (AR, residues 210–250); and C-terminal coiled-coil domain (Figure 2B). The N-terminal region appears to have an autoinhibitory effect to prevent HEXIM1−P-TEFb association in the absence of 7SK RNA (Michels et al., 2004; Li et al., 2005). In HEXIM1 proteins the ARM contains two basic segments, KKKHRRR and KKKRHWK, linked by a conserved PS sequence (Yik et al., 2004). The ARM binds to the SL1 distal region of 7SK RNA (Lebars et al., 2010). Phosphorylation of HEXIM1 ARM residue S_158_ by PKC abolishes interactions with 7SK RNA, likely through electrostatic repulsion (Fujinaga et al., 2012). As a dimer, HEXIM1 has up to two RNA binding sites. One well-established binding site is the GAUC2-GAUC3 motif on the distal 5′ SL1 (Figures 3, 4, 8) (Lebars et al., 2010). A second site has been identified at the proximal end of SL1 for the “linear” 7SK RNA model (Muniz et al., 2010). Although SL1 is remodeled in the “circular” conformation, SL1alt contains a single putative HEXIM1 binding site (GAUC1-GAUC2) (Figures 3D, F), and an RNA construct of SL1alt was able to bind HEXIM1 (Li et al., 2007).

D’Souza and coworkers determined four solution NMR structures of the 7SK RNA SL1 distal construct, used previously for structure determination, in complex with the first segment of the HEXIM1 ARM RNA-binding motif (residues 145–156) or Tat ARM variants (residues 44–60) (Figure 8A; Table 2). Tat and HEXIM1 ARM peptides bound in the major groove between ASM1 and ASM4, but did not bind to ASM motif base triples. Rather, ARM peptide residues were primarily found to interact with base-pairs adjacent to base triples, acting as a “sandwich” around the base triple, as well as to the phosphodiester backbone (Figures 8A, B). Peptide-bound structures showed wide variance in binding modes, potentially due to degeneracy in available hydrogen bonding sites within the major groove and backbone (Figure 8A). Tat ARM, which is enriched in arginine residues compared to HEXIM1 (Table 2), appears to have increased RNA interactions compared to HEXIM1 ARM particularly at the N-terminus (Figure 8A). Four arginine residues in Tat peptides (R_52_, R_53_, R_56_, R_57_) intercalate into the major groove and interact with ASM1, ASM2, ASM3, and ASM4; In contrast, only two arginine residues in HEXIM1 peptide (R_155_ and R_156_) interacted with ASM1 and ASM2 (Figure 8B). In binding assays, Tat peptide preferentially bound to SL1 distal compared to HEXIM1 peptide (Pham et al., 2018), consistent with previous studies showing that Tat could displace HEXIM1 from 7SK RNA (Muniz et al., 2010).

In addition to serving as a dimer interface, the C-terminal coiled-coil domain contains the CycT1 binding domain (TBD) (Dames et al., 2007). Structures have been determined of the HEXIM1 TBD domain by solution NMR and X-ray crystallography (Table 2). The solution NMR structure of the HEXIM1 TBD (residues 255–339) (Table 2) showed that the coiled-coil motif forms a dimer *via* leucine zipper (Figure 8C). In addition, a “stammer” motif (residues 315–318) was identified, which is a short unstructured linker that disrupts the formation of a continuous α-helix (Figure 8C). An X-ray crystal structure of the HEXIM1 TBD lacking the stammer motif (TBD-Δstammer, Δ316-318) showed that the coiled-coil motif forms a continuous α-helix and repositions the second coiled-coil segment (Figure 8C).

### 7SK RNP assembly with P-TEFb

While domain structures of HEXIM1 ARM or coiled-coil motifs have been determined, structural information on HEXIM1 binding to P-TEFb and recruitment to 7SK RNP are lacking. HEXIM1 domains are involved in binding both CycT1 and CDK9. The stammer motif in the C-terminal TBD was shown to be important for CycT1 binding, and deletion resulted in a 5-fold reduction in HEXIM1−CycT1 association compared to wild-type (Schonichen et al., 2010). A model of HEXIM1-mediated P-TEFb inhibition has been proposed in which on binding to 7SK RNA, the HEXIM1 PYNT site is exposed to enable the assembly of P-TEFb (Michels et al., 2004). HEXIM1 directly interacts with the T-loop in the CDK9 active site through the central PYNT motif, masking the substrate-binding site to inhibit kinase activity (Michels et al., 2004; Kobbi et al., 2016; Michels and Bensaude, 2018). Assembly of P-TEFb with 7SK RNP requires that CDK9 T-loop residue T_186_ is phosphorylated (Chen et al., 2004). Several phosphatases have been identified that dephosphorylate CDK9 T_186_ (Chen et al., 2008; Gudipaty et al., 2015) as part of the P-TEFb release mechanism Table 1. In order to have P-TEFb catalytic activity restored, CDK9 T_186_ must be re-phosphorylated upon P-TEFb release.

In addition to HEXIM1 requirement for P-TEFb assembly, 7SK SL4 and Larp7 are also required for stable association of P-TEFb onto 7SK RNP. In the HIV-1 TAR element, CycT1 and Tat cooperate to bind to the TAR apical loop (Schulze-Gahmen and Hurley, 2018). A similar interaction has been proposed for 7SK RNP, where CycT1-Tat interacts with SL4 (Durney and D'Souza, 2010). However, experimental evidence to support this model is lacking. Given the stable association of SL4 with Larp7 and MePCE, it is more likely that P-TEFb interacts with Larp7 rather than SL4. A C-terminal Larp7 construct (residues 375–589) was able to co-immunoprecipitate CDK9, supporting a Larp7−P-TEFb interaction (Markert et al., 2008). Although a direct interaction between CycT1 and 7SK RNA has not yet been demonstrated, it is possible that CycT1 may bind 7SK RNA in addition to HEXIM1 and Larp7.

## Conclusion and perspectives

Advances in 7SK RNP structural biology over the past decade have significantly improved our understanding of 7SK RNP assembly and maturation. A consensus model has started to develop that describes early 7SK RNA biogenesis. Likewise, a nucleotide-resolution model is beginning to emerge that describes 7SK RNA secondary structure switching in response to different functional states. High-resolution structures of individual domains, RNA-protein complexes, and ternary complexes have increased understanding of HEXIM1 recognition of 7SK RNA, as well as assembly into a stable 7SK core RNP. Rich biochemical data have provided insights into P-TEFb sequestration and kinase inactivation and have revealed alternate pathways of 7SK RNP function in RNAPII transcription regulation. However, despite these advances, several outstanding questions remain unresolved, particularly regarding the structural basis for P-TEFb assembly, kinase inactivation, and release.

What is the 7SK RNA secondary structural landscape, and how is 7SK RNA secondary structure remodeling achieved? Several secondary structure models have been proposed using biochemical or bioinformatics approaches. While some 7SK RNA regions have common secondary structures across models, residues spanning SL1-SL2 vary substantially. Several helicases have been loosely identified to bind to the central region of 7SK RNA (Table 1). It has been proposed that helicase recognition leads to secondary structure switching, enabling the assembly or release of downstream accessory proteins such as P-TEFb. However, at present there is little mechanistic or structural understanding of how these proteins alter 7SK RNA secondary structure. Recent chemical probing-based approaches have shown that 7SK RNA secondary structure differs based on protein composition (Flynn et al., 2016; Brogie and Price, 2017), and that 7SK RNA exists as an ensemble of multiple secondary structures (Luo et al., 2021; Olson et al., 2022). However, direct evidence connecting a specific secondary structure with a specific protein-bound state remains tenuous. More studies combining proteomics with chemical probing experiments are needed to validate the proposed secondary structural models and switching mechanism. It is plausible that 7SK RNA forms alternate secondary structures that are not yet accounted for, and it will be interesting to see how future studies continue to shape our understanding of the complexity of 7SK RNA structure. Beyond protein-mediated RNA structural dynamics, there is a growing acknowledgement of the impact of posttranscriptional modifications on RNA structure and function (Lewis et al., 2017). How these modifications (Table 1) impact 7SK RNA structure and protein recognition is unexplored territory. Finally, although progress has been made toward modeling 7SK RNA secondary structure, it remains unclear how RNA domains are organized in 3D space to enable protein recognition.

How is P-TEFb assembled onto 7SK RNP, and what is the mechanism of kinase inactivation and re-activation? A picture is starting to emerge regarding HEXIM1 recognition of 7SK RNA, and HEXIM1 recognition of P-TEFb. However, a lack of structural information has impeded detailed understanding of 7SK RNP sequestration and inactivation of P-TEFb. In addition to HEXIM1, evidence suggests that P-TEFb interacts with Larp7 (Markert et al., 2008). Moreover, 7SK RNA may potentially interact with P-TEFb to further promote stable association. Beyond P-TEFb, other components of the SEC may also associate with 7SK RNP (Table 1). When bound to 7SK RNP, P-TEFb is sequestered in a catalytically inactive state. HEXIM1 has been proposed to act as a steric block to prevent CDK9-substrate interactions. Several studies have shown that CDK9 phosphorylation status, particularly of active site residue T_186_, changes as part of the release mechanism from 7SK RNP. The basis for P-TEFb kinase inactivation, and re-activation upon 7SK RNP release, remains unclear. Additional studies are needed to gain an atomistic understanding of P-TEFb assembly with 7SK RNP, P-TEFb inactivation when bound to 7SK RNP, and re-activation upon release from 7SK RNP.

How do accessory proteins bind 7SK RNP to direct P-TEFb release and alternate functional pathways? Numerous proteins have been identified to be involved in P-TEFb release from 7SK RNP, including RNA processing factors, transcription factors, and helicases (Table 1). Rather than act as a universal transcriptional activator, it appears that specific cellular signals promote P-TEFb release and re-activation to stimulate expression of specific genes. 7SK RNP function appears to be particularly critical for regulating cell proliferation (Zhou and Yik, 2006; Musavi et al., 2019) and DNA damage response (Bugai et al., 2019; Lans et al., 2019; Studniarek et al., 2021). In addition, 7SK RNP appears to regulate RNAPII in multiple pathways. Intriguingly, 7SK RNP is found in different subcellular compartments in each of these pathways including the nucleolus (He et al., 2006), Cajal bodies (Egloff et al., 2017), nuclear speckles (Barboric et al., 2005; Prasanth et al., 2010; Ji et al., 2013), and cytoplasm (Briese et al., 2018; Ji et al., 2021). It is tempting to speculate that assembly of specific accessory proteins directs the 7SK RNP to different intracellular locations to carry out diverse functions.

Although substantial progress has been made in 7SK RNP structural biology, the sparsity of determined structures has remained an obstacle toward gaining a detailed understanding of how 7SK RNP assembles and functions in transcription regulation. The inherent structural plasticity of 7SK RNA and heterogeneity of 7SK RNP particles has posed a significant challenge toward structural elucidation. Overcoming these challenges will be critical to advance understanding of 7SK RNP structure, particularly for macromolecular complexes. Ultimately, structural biology coupled with biochemical, -omics, and cellular approaches will enable a comprehensive understanding of 7SK RNP cellular function and role in human health and disease.
